# Granulomatosis with polyangiitis with salivary glands involvement: Presenting a case and describing its clinical, pathophysiological, and therapeutic aspects

**DOI:** 10.1002/ccr3.7703

**Published:** 2023-07-13

**Authors:** Arman Ahmadzadeh, Faraneh Farsad, Neda Babadi, Dena Mohamadzadeh

**Affiliations:** ^1^ Rheumatology Ward of Loghman Hakim Hospital Shahid Beheshti University of Medical Sciences Tehran Iran; ^2^ Research Centre of Loghman Hakim Hospital Shahid Beheshti University of Medical Sciences Tehran Iran; ^3^ Department of Adult Rheumatology, Loghman Hakim Hospital, School of Medicine Shahid Beheshti University of Medical Sciences Tehran Iran; ^4^ Clinical Research Development Center, Imam Reza Hospital Kermanshah University of Medical Sciences Kermanshah Iran

**Keywords:** granulomatosis with polyangiitis, parotid gland, salivary glands, submandibular gland

## Abstract

Granulomatosis with polyangiitis (GPA), a rare form of small vessel vasculitis, may be manifested by multisystem involvement misleading its definitive diagnosis. The involvement of salivary glands is a very rare characteristic of GPA. Herein, we described a case of GPA with submandibular salivary gland involvement followed by reviewing the literature on similar cases. The case was a 31‐year‐old man, a known case of seronegative peripheral arthritis that referred recently with bilateral enlargement of the parotid and submandibular glands. Pulmonary nodules were also evident in the patient's CT scan. Fine‐needle aspiration under ultrasound guidance indicated the presence of degenerated squamoid cells, giant cells, and inflammatory cells with a priority of neutrophils in the submandibular gland, as well as the presence of a cyst containing fluid without the evidence of malignancy in the parotid gland. The positivity for the Anti‐neutrophil Cytoplasmic Antibody (C‐ANCA) marker was also revealed. The patient was treated with methotrexate, prednisolone, and rituximab which led to a gradual reduction in the size of the glands and the improvement of the patient's clinical symptoms within 1 month after the treatment. Enlargement of salivary glands in the context of inflammatory disorders can raise doubts about the existence of GPA, and therefore imaging evaluation and histopathological assessment with an ANCA test will be necessary to confirm or rule out it.

## 1 INTRODUCTION

Granulomatosis with polyangiitis (GPA) is a rare form of small vessel vasculitis characterized by multisystem necrotizing granulomatous lesions with different organs involvement such as the central nervous system, urinary tract, gastrointestinal tract, skin, and even eye.^1^, ^2^, ^3^ The main pathological characteristic of GPA is the positivity of autoantibodies against cytoplasmic components of neutrophil cells or C‐ANCA as well as proteinase‐3 (PR3) over‐activation.^4^ Due to the multisystem involvement nature, the diagnosis of GPA is difficult. In addition, GPA may overlap with other clinicopathological conditions such as infections, autoinflammatory disorders, connective tissue diseases, and even neoplasms.^5^, ^6^ In this regard, the involvement of salivary glands is a very rare phenomenon. Herein, we described a case of GPA with submandibular salivary gland involvement followed by reviewing the literature on similar cases.

## 2 CASE PRESENTATION

**FIGURE 1:**
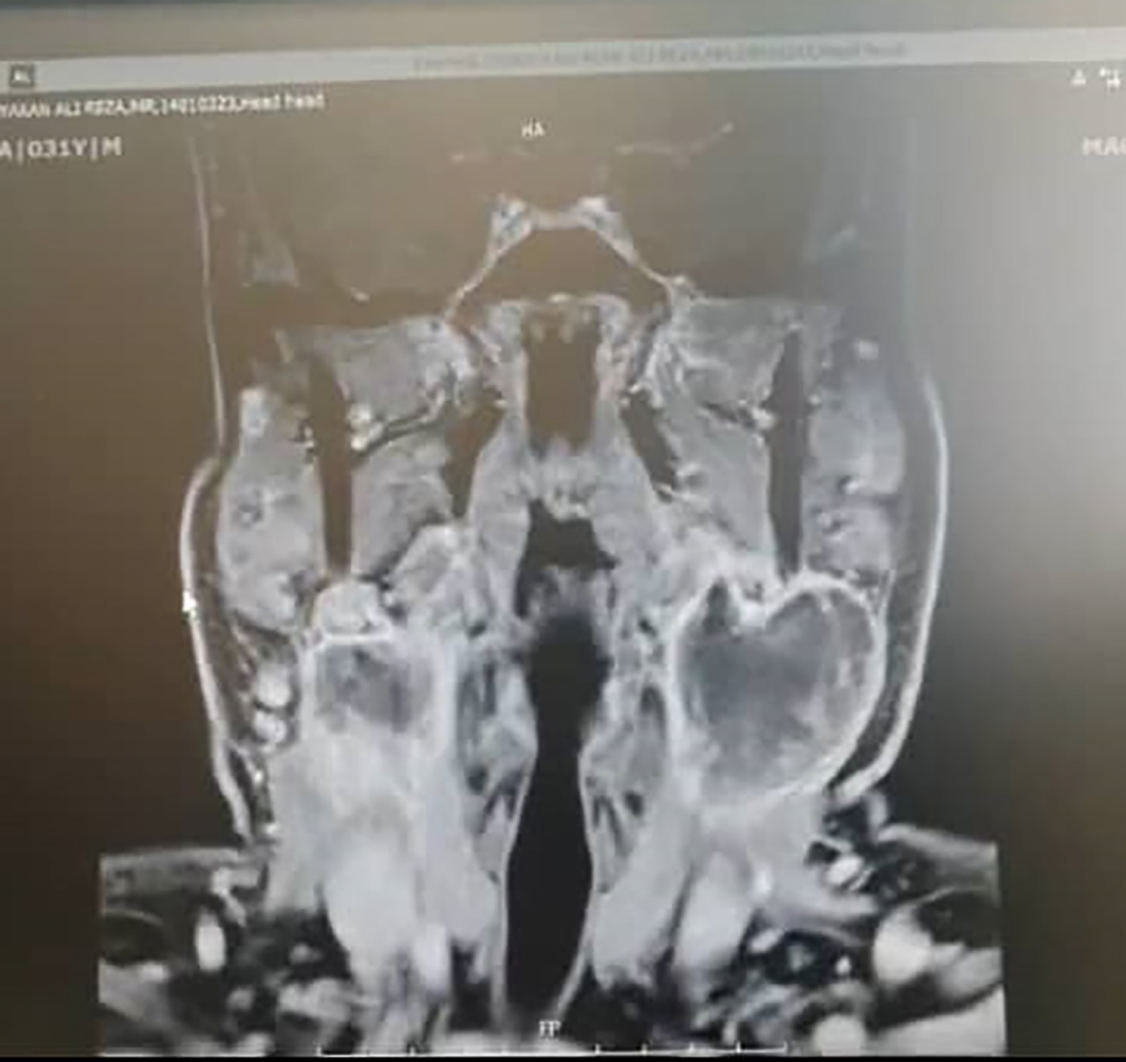
MRI view of parotid and submandibular glands.

**FIGURE 2:**
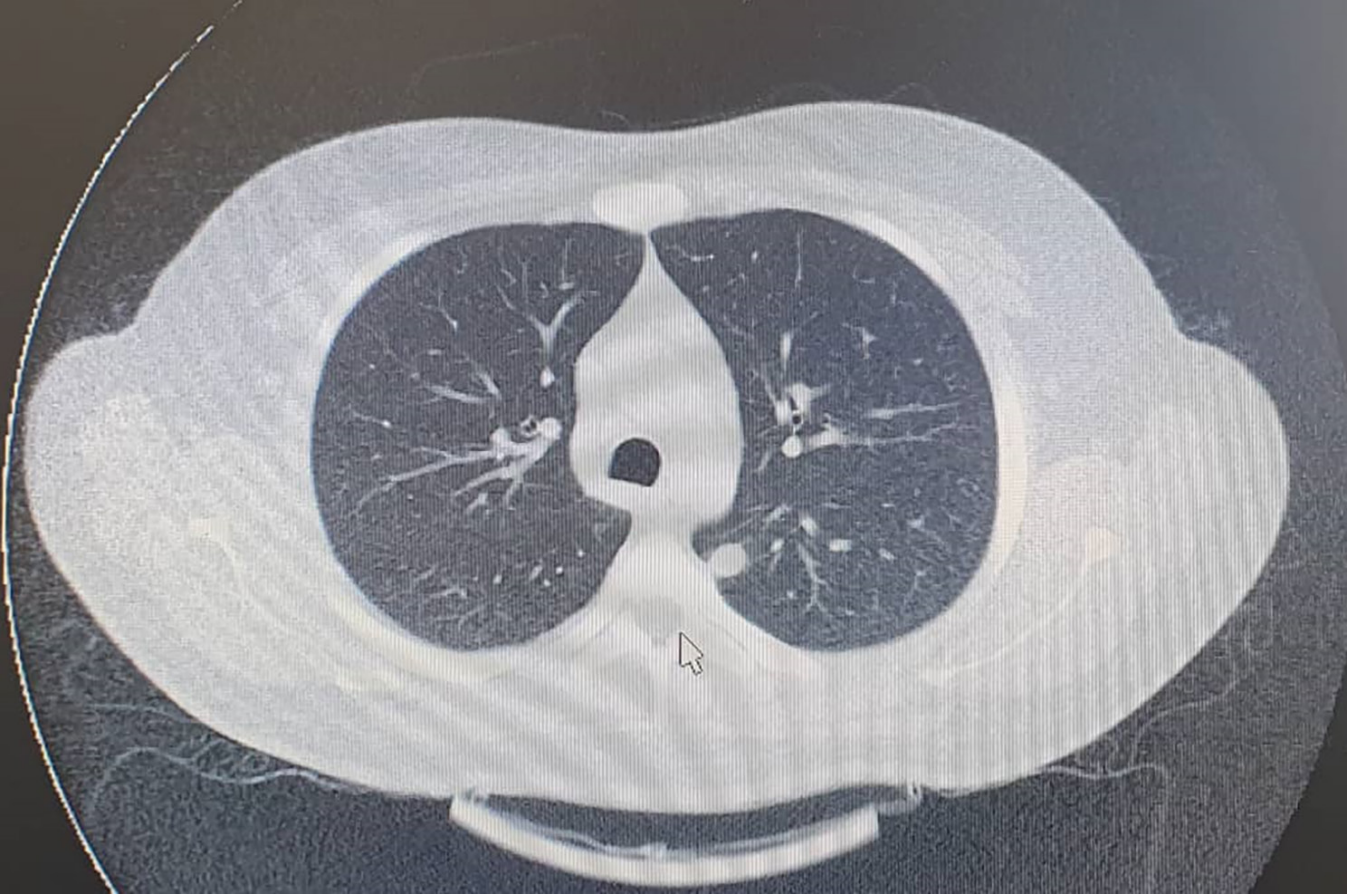
A feature of pulmonary nodules in CT scanning.

**FIGURE 3:**
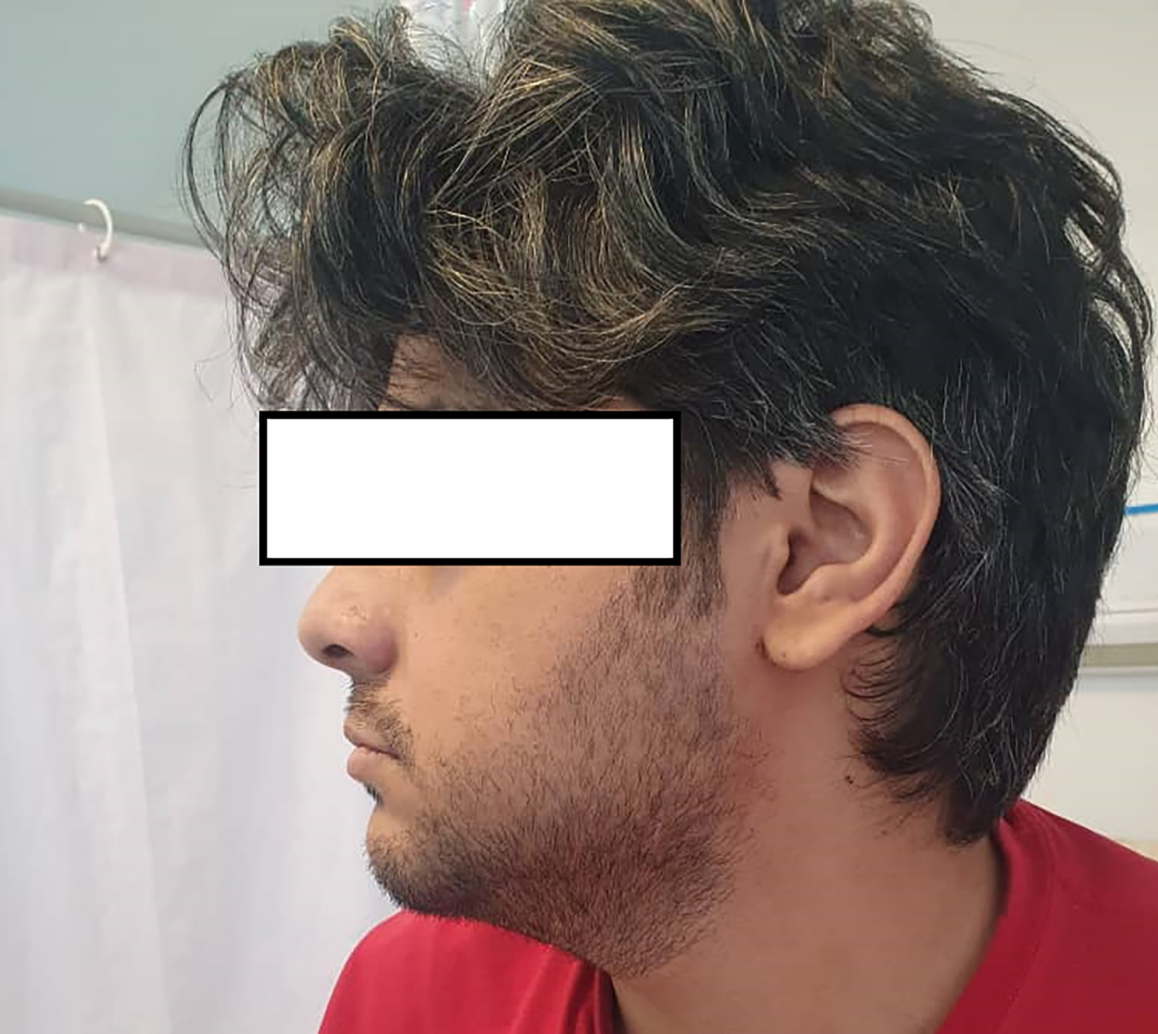
The improvement of the patient's clinical symptoms within 1 month after the treatment.

The case was a 31‐year‐old man, a known case of seronegative peripheral arthritis with involvement of knees, hand wrists, and metacarpophalangeal joints from 5 years ago that was treated with methotrexate 15 mg/weekly and prednisolone 5 mg/daily. During the years of illness, the patient had no evidence of skin nodules, exertional dyspnea, sinusitis, or kidney insufficiency, but evidence of scleritis was found during the course of the disease that improved with a gradual increase in the dose of the drug. About 6 months before the last visit, the patient voluntarily stopped his medication regimen, and after that, about 2 months before, he developed bilateral enlargement of the parotid and submandibular glands. At the time of the visit, the swelling of the mentioned areas was evident in the form of a thundering mass without erythema on the surface of the glands. On examination, the left mandibular margin was not well palpable. The patient did not complain of dry mouth, but he complained of dry eyes without any evidence of visual impairment, pain, or eye inflammation. There was no evidence of purulent or bloody discharge from the nose. Also, there were no signs of fever or shortness of breath. In initial laboratory assessment, cell count, serum biochemistry, thyroid test, and 24‐h urine analysis were found to be normal; however, raised inflammatory indices including C‐reactive protein and erythrocyte sedimentation rate were evident. The purified protein derivative skin test was also negative. In sonography assessment, a cystic mass with internal echogenic foci measuring 20 × 27 mm was seen in the distal part of the left submandibular gland. For this reason, the patient was a candidate for MRI with contrast indicating a cystic‐like lesion with fluid–fluid level and peripheral enhancement in parotid and submandibular glands bilaterally suggestive of hematoma (Figure 1). Pulmonary nodules were also evident in the patient's CT scan (Figure 2). There was also a severe enlargement of bilateral parotid and submandibular glands probably due to vasculitis. Based on the aforementioned findings, the patient underwent fine‐needle aspiration (FNA) under ultrasound guidance, which indicated the presence of degenerated squamoid cells, giant cells, and inflammatory cells with a priority of neutrophils in the submandibular gland, as well as the presence of a cyst containing fluid without the evidence of malignancy in the parotid gland. The differential diagnosis proposed for the patients included Sjogren's syndrome, sarcoidosis, IgG4‐related disease, and Wegener's granulomatosis (GPA). As additional evaluations, an ultrasound of the abdomen and pelvis was requested, the only positive finding of which was grade 1 fatty liver. CT scan of the sinuses was also normal. In the CT scan of the lung, a lung nodule with a diameter of 14 mm was seen in the medial apical segment of the lung, which was not seen in the CT scan of the patient 4 years ago. The echocardiography of the patient also showed a normal ventricular functional state with normal pulmonary artery pressure and mild MR. In cytological assessment, mildly raised serum levels of immunoglobulin‐G4 (IgG4) 2 g/L (normal<1.4 g/L) along with positivity for the C‐ANCA 500 AU/mL (normal<20 AU/mL) marker without any positivity for other rheumatologic markers were revealed. Also, the viral markers of hepatitis and HIV were found to be negative. Due to the size of the submandibular gland containing necrosis, the presence of pulmonary nodules, and positive markers, the diagnosis of GPA was finally made for the patient. He was treated with methotrexate 20 mg/weekly, prednisolone 1 mg/kg/daily with gradual tapering to 5 mg/daily, and rituximab 500 mg every week for four doses which led to a gradual reduction in the size of the glands and the improvement of the patient's clinical symptoms within 1 month after the treatment (Figure 3).

## 3 DISCUSSION

Wegener granulomatosis or GPA is one of the rare components of a wide spectrum of diseases named ANCA‐associated vasculitides. This disease was first described by Heinz Klinger and colleagues in 1931 those 5 years later; Friedrich Wegener described a series of affected patients and introduced it as a distinct form of vasculitis.^7^ From an etiological point of view, the exact etiologies of GPA remain unknown; however, the relationship between the pathophysiological changes of the disease and ANCA has been fully confirmed. Now, it seems that a variety of genetic and microbial factors involve in triggering disease and its severity. It is now presumed that the inflammatory basis of GPA is referred to as ANCA positivity.^8^ In pathological assessments, it has been shown that immune response to some environmental insults may result in hyper‐secretion of some cytokines such as tumor necrosis factor, interleukin 17, and interferon‐gamma leading ultimately to the development of granulomatous vascular lesions.^9^ Also, via interaction between ANCA and PR3 enzyme, the adhesion of neutrophils to vascular endothelium can be triggered which leads to damage to endothelial cells.^10^ Moreover, the over‐expression of some genes has been also described to be associated with GPA such as the CTLA‐4 gene (involves in T‐cell activation), PRTN‐3 (involves in activation of PR3), and HLA‐DP gene (involves in activation of neutrophils and monocytes).^11^ Regarding the etiologic role of infections, some evidence is available in the flaring role of hepatitis C virus, Epstein–Barr virus, cytomegalovirus, parvovirus, and COVID‐19 infections and the appearance of GPA.^12^, ^13^ Also, it is now suggested a close link between the likelihood of GPA and some medications such as phenytoin, hydralazine, allopurinol, and anti‐thyroid drugs with unclear etiological roles.^14^, ^15^ Epidemiologically, the annual incidence of GPA is estimated at 10–20 cases per one million directly dependent on geographical characteristics, with a higher incidence in colder areas. The disease is more prominent in older adults than in children whose age peaks at 64–75 years old but with no gender predilection.^16^

GPA is known as a multisystem syndrome with evidence of inflammatory reactions in different organs such as the upper respiratory tract (as rhinitis, sinusitis, otitis, or mastoiditis), vascular bed (as small vessels damages, vasculitis), kidney system (as glomerulonephritis), and respiratory system (as alveolar hemorrhage and lung nodules). In this regard, appearing generalized systemic symptoms, especially nonspecific symptoms, are expected in relation to GPA. Upper respiratory involvement is predictable in more than 90% of patients, lower respiratory tract involvement (as pulmonary infiltrations, pleural effusion, and nodules) in about 15%–50% of patients, renal involvement in 10%–20% of patients, eye defects (commonly as scleritis) in about 50%, skin defects (as purpura, nodules, ulcers, and granulomas) in 50%–60%, nervous system (commonly as peripheral neuropathies) in 30%–40%, musculoskeletal system (as myalgia and/or arthralgia) in 70%, and even cardiovascular system (as valvular lesions or pericarditis) in less than 10% of patients.^17^, ^18^, ^19^, ^20^

To evaluate patients suspected of GPA, all clinical and paraclinical assessments should be considered including minute physical examination, tracking laboratory parameters including blood count, electrolytes, inflammatory markers, imaging, and tittering specific markers of PR3‐ANCA and histological assessments if required.^21^ Radiological assessment according to the involved organs should be also proposed. Such management can help to differentiate GPA from other misleading conditions. With respect to therapeutic approaches, the treatment of such patients is based on the combination of immunosuppressive drugs such as glucocorticoids, cyclophosphamide, methotrexate, and rituximab.^22^ In more severe cases, plasmapheresis may be also indicated. In life‐threatening conditions, a combination of glucocorticoids and cyclophosphamide was found to be a very effective regimen.^23^ The efficacy and safety of rituximab and according to the RAVE trial have demonstrated the similarity of effectiveness and safety of this drug compared to cyclophosphamide.^24^ In GPA with a milder state, glucocorticoids combined with methotrexate are preferred. In those with renal dysfunction or pulmonary hemorrhage complicated by respiratory compromise, plasmapheresis should be considered.^25^

GPA with salivary glands involvement has been reported rarely. In the present case, despite the previous evidence of arthritis, the occurrence of swelling and involvement of the parotid and submandibular glands along with the observation of the pulmonary nodule raised the suspicion of the occurrence of other autoimmune disorders. Based on this, the general and specific evaluations indicated the positivity of the ANCA marker in this patient, which, along with pulmonary nodular involvement, strongly suggested GPA. Thus, the treatment with a combination of methotrexate, prednisolone, and rituximab was selected leading to improvement of clinical condition. IgG4‐related disease was the main differential diagnosis of our case. As we mentioned in the case presentation section FNA of the salivary glands showed degenerated squamoid cells, giant cells, and inflammatory cells with a priority of neutrophils in the submandibular gland, and as we know the key histopathology characteristics of IgG4‐related disease are dense lymphoplasmacytic infiltrate organized in a storiform pattern which tends to aggregate around ductal structures. Lymphoid follicles and germinal centers are common. The important diagnostic clues on FNA to suspect a diagnosis of IgG4‐RD include low cellularity despite adequate effort, inflammatory background rich in lymphocytes, and spindle cells admixed with a few plasma cells and eosinophils.^26^ Therefore, the histological findings were not compatible with IgG4‐RD. And as we know a mildly increased serum level of IgG4 might be found in GPA.^27^ But a highly increased level of C‐ANCA confirmed the diagnosis of GPA in our case. In a similar study by Ryota Kikuchi et al,^28^ the case described was an old man manifesting with low‐grade fever and painful enlargement of the right submandibular gland that led to the definitive diagnosis of GPA based on the presence of multiple organs involvement along with histopathologic evidence in a skin biopsy sample of necrotizing granulomatous inflammation with vasculitis. He was successfully treated with methylprednisone and cyclophosphamide with gradual improvements in symptoms and radiologic findings. Alper Ceylan et al.^29^ described a young woman with parotid gland swelling along with cough, arthralgia, epistaxis, nasal obstruction, weight loss, and resistance to antibiotic therapy. The prominent findings, in this case, were to reveal diffuse enlargement and cutaneous fistulas on the bilateral parotid glands and also positivity for c‐ANCA that the diagnosis was strongly in favor of the disease. In general, it should be kept in mind that one of the diagnoses in case of symptoms of inflammatory reaction in salivary glands along with involvement in other vital organs such as lung nodular involvement should be this disease and its treatment management should be based on the treatment protocols as soon as possible.

## AUTHOR CONTRIBUTIONS

**Arman Ahmadzadeh:** Conceptualization; writing – review and editing. **Faraneh Farsad:** Writing – original draft. **Neda Babadi:** Writing – original draft. **Dena Mohamadzadeh:** Data curation.

## FUNDING INFORMATION

We received no funding.

## CONFLICT OF INTEREST STATEMENT

The authors declare that they have no competing interests.

## ETHICS STATEMENT

Approval was not needed by the local Clinical Research Ethics Committee for case reports.

## CONSENT STATEMENT

Written informed consent was obtained from the patient for publication of this case report and any accompanying images. A copy of the written consent is available for review by the Editor‐in‐Chief of this journal.

## Data Availability

Data are available if requested.
